# Long-Term Outcomes on Antiretroviral Therapy in a Large Scale-Up Program in Nigeria

**DOI:** 10.1371/journal.pone.0164030

**Published:** 2016-10-20

**Authors:** Seema T. Meloni, Charlotte A. Chang, Geoffrey Eisen, Toyin Jolayemi, Bolanle Banigbe, Prosper I. Okonkwo, Phyllis J. Kanki

**Affiliations:** 1 Department of Immunology and Infectious Diseases, Harvard T.H. Chan School of Public Health, Boston, Massachusetts, United States of America; 2 Center for Global Health, Northwestern University Feinberg School of Medicine, Chicago, Illinois, United States of America; 3 AIDS Prevention Initiative in Nigeria, Limited by Guarantee, Abuja, Federal Capital Territory, Nigeria; CEA, FRANCE

## Abstract

**Background:**

While there has been a rapid global scale-up of antiretroviral therapy programs over the past decade, there are limited data on long-term outcomes from large cohorts in resource-constrained settings. Our objective in this evaluation was to measure multiple outcomes during first-line antiretroviral therapy in a large treatment program in Nigeria.

**Methods:**

We conducted a retrospective multi-site program evaluation of adult patients (age ≥15 years) initiating antiretroviral therapy between June 2004 and February 2012 in Nigeria. The baseline characteristics of patients were described and longitudinal analyses using primary endpoints of immunologic recovery, virologic rebound, treatment failure and long-term adherence patterns were conducted.

**Results:**

Of 70,002 patients, 65.2% were female and median age was 35 (IQR: 29–41) years; 54.7% were started on a zidovudine-containing and 40% on a tenofovir-containing first-line regimen. Median CD4+ cell counts for the cohort started at 149 cells/mm^3^ (IQR: 78–220) and increased over duration of ART. Of the 70,002 patients, 1.8% were reported as having died, 30.1% were lost to follow-up, and 0.1% withdrew from treatment. Overall, of those patients retained and with viral load data, 85.4% achieved viral suppression, with 69.3% achieving suppression by month 6. Of 30,792 patients evaluated for virologic failure, 24.4% met criteria for failure and of 45,130 evaluated for immunologic failure, 34.0% met criteria for immunologic failure, with immunologic criteria poorly predicting virologic failure. In adjusted analyses, older age, ART regimen, lower CD4+ cell count, higher viral load, and inadequate adherence were all predictors of virologic failure. Predictors of immunologic failure differed slightly, with age no longer predictive, but female sex as protective; additionally, higher baseline CD4+ cell count was also predictive of failure. Evaluation of long-term adherence patterns revealed that the majority of patients retained through 84 months maintained ≥95% adherence.

**Conclusion:**

While improved access to HIV care and treatment remains a challenge in Nigeria, our study shows that a high quality of care was achieved as evidenced by strong long-term clinical, immunologic and virologic outcomes.

## Introduction

The rapid scale-up of global HIV antiretroviral therapy (ART) in resource-constrained settings (RCS) over the past decade has successfully enrolled millions of HIV-infected patients in care and treatment programs [1]. While the initial goal of these programs was to initiate large numbers of patients on ART and, subsequently, reduce overall morbidity and mortality, the continuing aim is to maintain patients on high quality life-long care. Many studies have examined short- and medium-term outcomes in adult patients enrolled in ART programs across the globe, but there are relatively limited data on the long-term outcomes for large-scale ART programs [2–12].

Nigeria is the most populous country in sub-Saharan Africa with an estimated population of nearly 180 million and current estimated HIV prevalence of 3.2%. Despite a low HIV prevalence, Nigeria has the second highest burden of HIV infection in the world [1,13,14]. In 2014, it was estimated that about 3.4 million people were living with HIV, with approximately 230,000 new HIV infections, representing almost 10% of the global HIV pandemic [13,15].

In 2001, the Federal Government of Nigeria initiated a national ART program as part of its enhanced response for the care and support for HIV-infected persons [16]. The Nigerian National ART program was initially rolled out to 25 designated ART centers distributed across the country’s six geopolitical zones. In collaboration with the Nigerian National ART Program, which had initiated treatment for over 13,000 HIV patients by mid-2004, the Harvard T. H. Chan School of Public Health (Harvard Chan) and Nigerian collaborators at the AIDS Prevention Initiative in Nigeria (APIN) initiated a rapid scale-up of HIV care and treatment programs through support from a PEPFAR grant beginning in 2004. The significant contribution of the PEPFAR program to the national ART program is apparent in the nearly exponential increase in patients initiated on ART between 2004 and 2012, with PEPFAR providing over 50% of the funding support for the scale-up. Over that same time period, national HIV prevalence estimates decreased from 3.8% [3.4%-4.1%] in 2005 to 3.2% [3.0%-3.5%] in 2013 [1]. From 2009 to 2012, the number of patients on ART in Nigeria rose from 303,000 to 491,000, and continued to increase to over 747,000 in 2014 [13].

Between 2004–2012, the Harvard/APIN PEPFAR program expanded from 6 to 36 hospitals and clinics, including 9 tertiary referral hospitals, 23 secondary hospitals or primary health clinics, and four non-governmental organizations (NGOs) in 9 of the 36 states of Nigeria (Benue, Borno, Enugu, Kaduna, Lagos, Ogun, Oyo, Plateau, and Yobe). Standardized protocols were developed for clinical management, laboratory testing and pharmacy handling, conforming to an optimized standard of care consistent with Nigerian National ART and PMTCT guidelines. Expanded and renovated clinics, pharmacies and equipped laboratories allowed for the provision of ART to large numbers of patients [17]. Computerized data entry of patients’ clinical, laboratory and pharmacy records was developed and implemented, making it possible to monitor the progress at the sites electronically [18]. As of March 2012, approximately 100,000 adult patients had received ART and 160,000 had received some form of HIV-related care. In addition, nearly 400,000 women had been provided prevention of mother-to-child transmission (PMTCT) services, with 20,000 mothers and children receiving the intervention.

In this evaluation, we provide data on long-term outcomes of adult patients enrolled in the Harvard/APIN PEPFAR Program between 2004–2012. A main objective of the study was to describe the baseline characteristics of the adult patients treated in the Harvard/APIN PEPFAR program and any changes in baseline characteristics over the 8-year study period. The other major objective of the study was to assess the long-term outcomes of ART, including immunologic and virologic. Additionally, we examined long-term adherence patterns for patients retained on ART.

## Methods

### Patient Information

The Harvard/APIN PEPFAR adult ART program enrolled patients with documented evidence of HIV infection by rapid test screening and HIV immunoblot confirmation. Since the Government of Nigeria commenced their ART program in 2002, the cohort included patients that might have already had as much as 2 years of previous ART at the time that they were enrolled in the Harvard/APIN PEPFAR program. For ARV-naïve patients, eligibility for ART in the Harvard/APIN PEPFAR program followed the Nigerian National Guidelines [19,20], which closely followed the World Health Organization (WHO) Guidelines at the time of patient enrollment [21–23]. Starting in 2004, patients were considered eligible for ART if their CD4+ cell counts dropped below 200 cells/mm^3^ or if symptomatic with CD4+ cell counts below 350 cells/mm^3^; criteria shifted to include CD4+ cell counts below 350 cells/mm^3^ regardless of symptoms starting in 2010. Written informed consent was obtained from all patients upon initial enrollment; for minors under 18 years of age, written informed consent from the parent/legal guardian and written assent to participate from the minor were dually documented. The protocol and consent forms were reviewed and approved by the Harvard Chan Institutional Review Board (IRB) and the Nigerian Institute for Medical Research IRB, which is a Federal-wide Assurance (FWA)-approved IRB that covers all other Harvard/APIN PEPFAR program sites.

We conducted a retrospective evaluation of the prospectively collected data for patients who were provided routine ART services through the Harvard/APIN PEPFAR Program and consented to participation in future evaluations. The analyses on long-term outcomes included patients who were enrolled on ART between June 2004-February 2012. All patients were at least 15 years of age at enrollment. Patients who had HIV-2 or dual HIV-1/2 infections, or were on a non-standard first-line (1L) ART regimen (i.e, not containing two nucleoside reverse transcriptase inhibitors [NRTI] plus one non-nucleoside reverse transcriptase inhibitor [NNRTI]) were excluded from the long-term outcomes analyses. Additionally, some issues in early viral load (VL) testing procedures and pharmacy documentation issues at two of our sites were discovered; therefore, patients who enrolled at either of those two sites before April 2007 were excluded from the evaluations as some of their early data were not reliable.

### Treatment

In early 2000, the production of generic ART drugs was just beginning and formed the basis of many ART programs in developing countries, including Nigeria. At PEPFAR program initiation in Nigeria, the most common 1L ART included stavudine (d4T), lamivudine (3TC), and nevirapine (NVP). In late 2006, the increased recognition of the toxicity and inferior efficacy of regimens containing d4T prompted the revision of international guidelines, with eventual removal of d4T from recommended 1L regimens. In 2003–2005, access to tenofovir (TDF)-based regimens was spreading to RCS, although the cost of the branded version slowed its introduction. In 2008–2009, the introduction of the generic equivalents and the fixed-dose combination (FDC) Atripla, which was a combination of TDF, emtricitabine (FTC), and efavirenz (EFV), expanded usage of TDF. In the Harvard/APIN PEPFAR program, TDF-based regimens were considered for patients with hepatitis B virus (HBV) co-infection and those with anemia; NVP was the NNRTI of choice in women of child bearing potential, due to the teratogenic potential of EFV; and, in cases of TB-HIV co-infection, patients were switched to EFV at higher dosing of 600 or 800 mg/day.

### Laboratory Measurements

Patient samples were drawn for laboratory testing at baseline, 6 months post-initiation of ART and every 6 months thereafter, regardless of prior treatment status. Laboratory tests included automated hematology, clinical chemistries, CD4+ cell counts by flow cytometry (CyFlow®, Partec, Munster, Germany), and plasma VLs using the COBAS® Amplicor version 1.5 (Roche Diagnostics, Rotkreuz, Switzerland). Hematology consisted of a complete blood count and clinical chemistries. Additional chemistry evaluations could be requested based on the physician’s discretion. Serology for HBV (Monolisa HBsAg Ultra3; Bio-Rad, Hercules, CA, USA) and hepatitis C virus (HCV) (Dia.Pro Diagnostic Bioprobes srl, Milan, Italy) infections was conducted at baseline. All patients were evaluated for tuberculosis (TB) using sputum and/or chest radiograph at baseline and at all subsequent clinical visits if suspected clinically [24]. Patients were switched to 2L regimens either based on virologic or clinical criteria, per Nigerian National Guidelines [19,20].

### Data Management

From the start of the Harvard/APIN PEPFAR program, all patient demographic, clinical and laboratory data were electronically captured using a Harvard Chan School-designed FileMaker Pro (FileMaker Pro, Santa Clara, CA) electronic medical record system (EMRS) [18]. Data forms were checked for errors prior to entry by data entry staff and entered into the EMRS, which had built-in error checks, including checks for laboratory values that were outside the ranges of acceptable values and flags for missing values. Data entry staff checked their records daily and then transferred files to data managers for weekly checks. Additional information regarding the structure of the EMRS were previously presented by Chaplin et al [18].

### Definitions

We evaluated baseline demographic (age, sex, education, employment status, marital status, HIV transmission risk category, enrollment year and enrollment site type) and clinical (ART regimen, WHO clinical stage, TB co-infection, HBV and/or HCV co-infection, body mass index (BMI), CD4+ cell count, VL and anemia) factors, where baseline was defined as the time of ART initiation. Baseline clinical assessments or laboratory evaluations for naïve patients were the closest measurements to, and up to six months before or 0.5 month after, their first ART pick-up date.

For analyses, age was converted to a categorical variable based on quartiles and occupation was collapsed into non-income generating (i.e., unemployed, students, job applicants, housewives/homemakers, and retirees) and income generating (laborers, service, and administrative support professionals vs. manager and other professional) categories. Clinical variables were collapsed into categories based on relevant thresholds for the regression. BMI was grouped into three WHO-defined categories: underweight (<18.5 kg/m^2^), normal (18.5–24.9 kg/m^2^), overweight (≥25.0). Anemia was defined using WHO-recommended hemoglobin cut-offs: non-anemia (≥12 g/dL for women and ≥13 g/dL for men), mild/moderate anemia (8–11.9 g/dL for women and 8–12.9 g/dL for men), and severe anemia (<8 g/dL; pregnancy was not differentiated). VL at baseline was stratified into three categories (≤10,000 copies/mL, 10,001–100,000 cp/mL, and >100,000 cp/mL). Virologic suppression was defined as a single viral load that was below the limit of detection (≤400 copies/mL). Using pharmacy prescription electronic refill records, which have been shown to be a valid proxy [25,26], average percent ART adherence was calculated as number of days supplied over total days in the given time interval, adjusting for leftover medication. Adherence categories were based on previously published conventions: ≥95%, 80–94.9%, and <80% [11,27–30].

Patients were classified as lost to follow-up (LTFU) if they had missed the last scheduled appointment by more than two months by the time of database closure. Patients for whom death, withdrawal, or transfer to non-Harvard/APIN sites was recorded during the period of evaluation were not considered LTFU. Data on deaths, withdrawals, or transfers were passively obtained when either patients or their acquaintances provided the information; active tracing of patients that were late to appointments was not feasible as part of the program.

Virologic failure (VF) was evaluated only for patients on ART ≥5.5 months (to provide window of time to include VLs close to month 6) and with ≥2 VLs after 6 months, and was defined as two consecutive VLs after 6 months on ART, which surpassed 1,000 copies/mL. Immunologic failure (IF) was evaluated for patients on ART ≥5.5 months and with ≥1 CD4+ cell count after 6 months and was defined using the following criteria: 1) CD4+ count <100 cells/mm^3^ after 6 months of treatment; 2) CD4+ count below baseline level after 6 months of treatment; and/or, 3) CD4+ count less than 50% of peak on treatment level. Patients that were switched to 2L ART were also considered virologic and immunologic failures. Patients for whom an alternate 1L regimen was substituted due to toxicity, stock-outs of certain medications, or other related issues were still considered to be on 1L and were not included in the failure categories.

### Statistical Analysis

Baseline demographics and clinical characteristics were measured using standard descriptive statistical methods. Bivariate comparisons of categorical variables were performed using the *Χ*^2^ test. A non-parametric test for trend was utilized for examining patterns in baseline CD4+ cell counts by enrollment year. Statistical significance was defined at an α-level of 0.05.

Kaplan-Meier survival analyses were used to estimate the time from the initiation of ART to two different major outcomes: 1) virologic failure; and, 2) immunologic failure. For patients who did not reach the endpoint, the data were censored at the date of the last visit. The log-rank test was used to compare survival times between strata for categorical variables. All predictors that were significant at the p<0.20 level in bivariate methods were further evaluated in a random effects Cox proportional hazards model, which accounted for heterogeneity between sites and was fitted using backward elimination. Additionally, we incorporated time-dependent factors into the analyses, including BMI, CD4+ cell count, VL, anemia status, and average percent adherence, which were measured every six months. VF during a given time period was assessed for associations with time-dependent factors measured during the previous time period. Since VF is defined as occurring at the first of two consecutive VLs >1000 cp/mL, any recorded VL during the time period immediately preceding VF would generally be suppressed; therefore, in the VF analysis only, we assessed unsuppressed VL during the current time period as a time-dependent predictor of VF, i.e., of a second consecutive unsuppressed VL. To address potential bias due to patients who were excluded because of missing data, multiple imputation of missing values was performed, following exploration of pattern of missigness and verification that data were missing at random, using chained equations assuming missing at random and 10 imputed data sets. Values for the Cox models were generated using both complete cases and multiply imputed data.

All statistical analyses were conducted using Stata version 13.1 (Stata Corporation, College Station, Texas, USA).

## Results

### Overview

**Fig 1:**
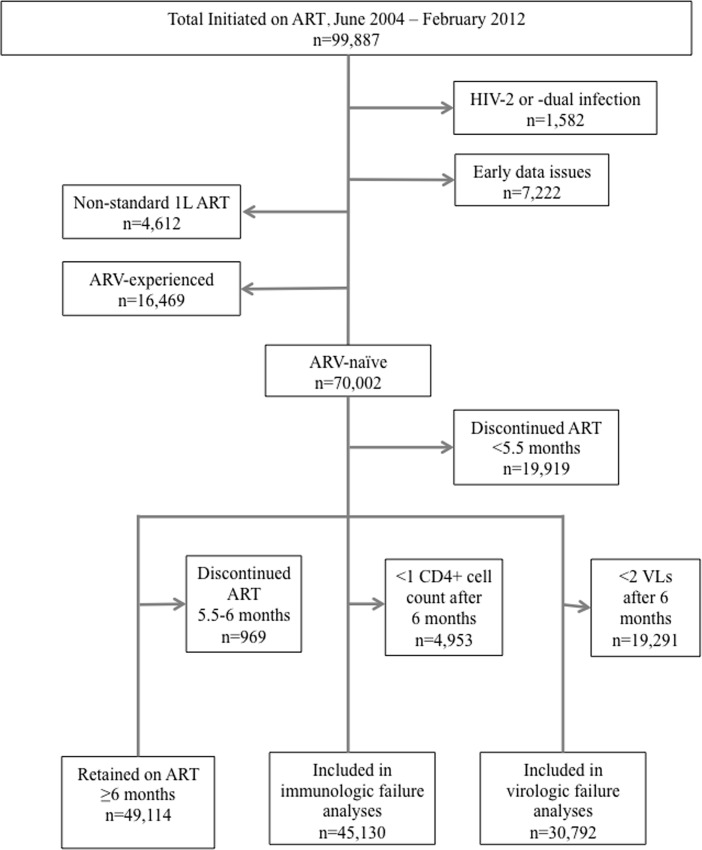
Flowchart highlighting enrolled patient population.

From June 2004 through February 2012, 99,887 adult patients were enrolled in the Harvard/APIN PEPFAR Program; of those patients, 91,083 were mono-infected with HIV-1 and had reliably collected data in the program EMRS. Within that subset, 86,471 initiated standard 1L regimens with 70,002 (81.0%) of those patients ARV-naïve and 16,469 (19.0%) with previous ARV experience at initiation in the Harvard/APIN PEPFAR program (Fig 1). The median follow-up period for all HIV-1-infected patients initiated on standard 1L regimens was 21.3 months (interquartile range; IQR: 5.2–41.3), with a range of 0–91.9 months. The median for those that were ARV-naïve at enrollment was 17.9 months (IQR: 4.0–38.3; range 0–87.8) and 36.0 months (IQR: 15.5–49.8) for those that were ARV-experienced.

### ARV-Naïve Patients

**Fig 2:**
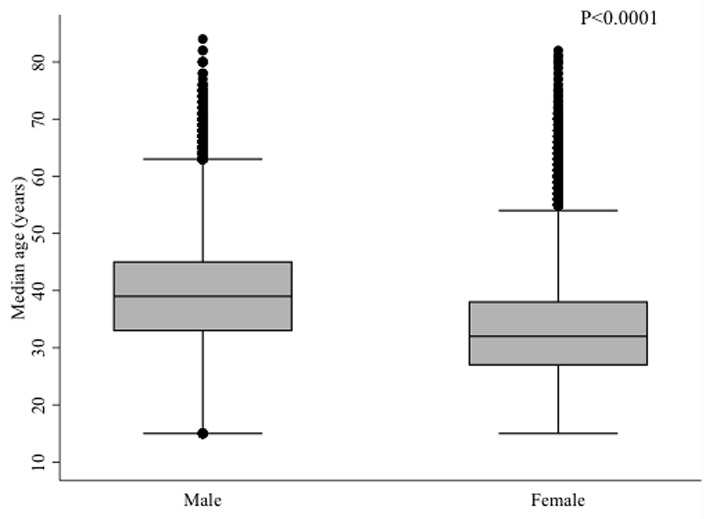
Median age at ART initiation by sex, ARV-naïve patients (N = 70,002).

Collectively, these 70,002 patients represent 136,852 (95% CI: 135,909–137,794) person-years (PY) on ART. Amongst the ARV-naïve patients enrolled on one of the standard 1L regimens, the majority were women (65.2%). The median age at entry was 35 years (IQR: 29–41), and was higher for males, at 39 years (IQR: 33–45), than for females, at 32 years (IQR: 27–38; p<0.0001; Fig 2).

**Fig 3:**
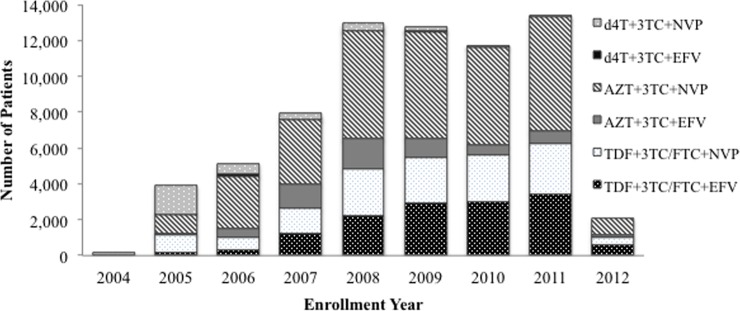
First-line ART regimen by enrollment year, ARV-naïve patients (N = 70,002).

The largest proportion of patients were initiated on AZT+3TC+NVP (46.1%; Table 1), but the proportions of patients initiating ART on the 6 standard 1L regimens changed significantly over the years of observation (Fig 3). The only regimen prescribed in 2004 was d4T+3TC+NVP, but that regimen was eventually phased out after AZT- and TDF-containing regimens were introduced in 2005. There was a large shift to AZT-containing regimens in 2006 and gradual shift to a more equal balance between AZT- and TDF-containing regimens by 2010.

**Table 1:** Baseline demographic and clinical characteristics.

	All Patients	ARV-naïve*	ARV-experienced*
Characteristic	n = 86,471	n = 70,002	n = 16,469
Time on ART**			
Range, months	0–91.9	0–87.8	0–91.9
Median, months (IQR)	21.3 (5.2–41.3)	17.9 (4.0–38.3)	36.0 (15.5–49.8)
**Age, years**			
15–29	22,125 (25.6)	18,855 (26.9)	3,270 (19.9)
30–34	18,394 (22.4)	15,830 (22.6)	3,564 (21.6)
35–40	20,323 (23.5)	16,249 (23.2)	4,074 (24.7)
≥41	24,549 (28.4)	18,982 (27.1)	5,547 (33.7)
*Median (IQR)*	*35 (29–42)*	*35 (29–41)*	*36 (31–43)*
**Female Sex**	56,386 (65.2)	45,605 (65.2)	10,781 (65.5)
**Education**			
None	15,307 (17.7)	12,993 (18.6)	2,314 (14.1)
Primary	18,057 (20.9)	15,225 (21.8)	2,832 (17.2)
Secondary	29,298 (33.9)	24,428 (34.9)	4,870 (29.6)
Tertiary	21,159 (24.5)	16,054 (22.9)	5,105 (31.0)
**Employment Status**			
Non-income-generating	20,248 (23.4)	16,509 (23.6)	3,739 (22.7)
Laborers, service, administrators	58,955 (68.2)	48,193 (68.9)	10,762 (65.3)
Professional, managerial	5,673 (6.6)	4,141 (5.9)	1,532 (9.3)
**Marital Status**			
Single	16,340 (18.9)	13,494 (19.3)	2,846 (17.3)
Married	49,063 (56.7)	40,038 (57.2)	9,025 (54.8)
Divorced/Separated	7,419 (8.6)	6,233 (8.9)	1,186 (7.2)
Widowed	12,425 (14.4)	9,334 (13.3)	3,091 (18.8)
**Heterosexual Sex as Risk Factor**	76,105 (88.0)	61,879 (88.4)	14,226 (86.4)
**ART Enrollment Year**			
2004–2006	12,834 (14.8)	9,272 (13.3)	3,562 (21.6)
2007–2009	43,645 (50.5)	33,677 (48.1)	9,968 (60.5)
2010–2012	29,922 (34.7)	27,053 (38.6)	2,939 (17.9)
**Tertiary Site**	74,207 (85.8)	58,676 (83.8)	15,531 (94.3)
**First-Line ART Regimen**			
TDF-3TC/FTC-EFV	14,755 (5.4)	13,865 (19.8)	890 (5.4)
TDF-3TC/FTC-NVP	15,296 (17.7)	14,160 (20.2)	1,136 (6.9)
AZT-3TC-EFV	6,809 (7.9)	6,043 (8.6)	766 (4.7)
AZT-3TC-NVP	38,561 (44.6)	32,297 (46.1)	6,264 (38.0)
d4T-3TC-EFV	440 (0.5)	286 (0.4)	154 (0.9)
d4T-3TC-NVP	10,610 (12.3)	3,351 (4.8)	7,259 (44.1)
**WHO Stage at baseline**			
1	19,772 (22.9)	15,164 (21.7)	4,076 (24.7)
2	21,705 (25.1)	17,066 (24.4)	3,744 (22.7)
3	23,055 (26.7)	19,086 (27.3)	3,315 (20.1)
4	8,830 (10.2)	7,780 (11.1)	759 (4.6)
**TB at baseline**	16,764 (19.4)	14,253 (20.4)	2,511 (15.2)
**HBV Status at baseline**	8,696 (10.1)	7,157 (10.2)	1,539 (9.3)
**HCV Status at baseline**	3,058 (3.5)	2,531 (3.6)	527 (3.2)
**BMI at baseline, kg/m**^**2**^			
Underweight (<18.50)	11,371 (13.2)	10,283 (14.7)	1,088 (6.6)
Normal (18.50–24.99)	37,357 (43.2)	29,702 (42.4)	7,655 (46.5)
Overweight (25.00–29.99)	10,900 (12.6)	7,390 (10.6)	3,510 (21.3)
Obese (≥30.00)	3,351 (3.9)	2,177 (3.1)	1,174 (7.1)
**Severe anemia at baseline**	6,771 (7.8)	6,238 (8.9)	533 (3.2)
**CD4 count at baseline, cells/mm**^**3**^			
≤100	23,176 (26.8)	21,516 (30.7)	1,660 (10.1)
101–200	26,378 (30.5)	23,863 (34.1)	2,515 (15.3)
201–350	21,232 (24.6)	17,249 (24.6)	3,983 (24.2)
>350	6,419 (10.7)	3,059 (4.4)	6,207 (37.7)
*Median (IQR)*	*166 (88–254)*	*149 (78–220)*	*312 (182–491)*
**Viral load at baseline, copies/mL**			
≤10,000	21,489 (24.9)	12,260 (17.5)	9,229 (56.0)
10,001–100,000	20,924 (24.2)	18,955 (27.1)	1,969 (12.0)
>100,000	25,527 (29.5)	24,180 (34.5)	1,347 (8.2)
*Median*	*45*,*730 (200–260*,*000)*	*71*,*310 (13*,*293–260*,*000)*	*486 (200–12*,*037)*

* Values shown are n and % unless otherwise indicated; % account for missing data

**Time on ART in the Harvard/APIN PEPFAR Program; does not account for time ART prior to entry for those with previous ARV experience and/or time on ART in another program for those that have transferred; value of 0 in the range refers to patients that made only 1 pharmacy visit and did not return, therefore no months of follow-up.

**Fig 4:**
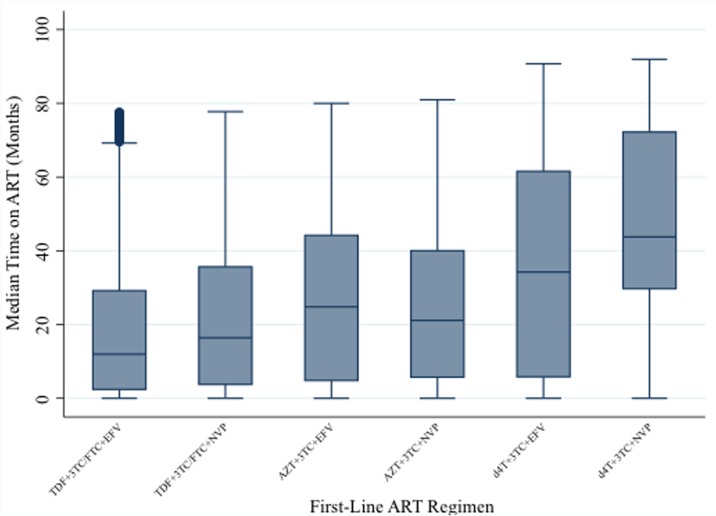
Median follow-up time on ART by first-line ART regimen, ARV-naïve patients (N = 70,002).

The median follow-up for patients who started on the different regimens were as follows: 31.7 months (IQR: 2.9–57.9; range: 0–79.8) for d4T+3TC+EFV; 50.6 (IQR: 14.7–77.0; range: 0–87.8) for d4T+3TC+NVP; 24.0 months (IQR: 4.6–43.9; range: 0–80.0) for AZT+3TC+EFV; 19.3 months (IQR: 4.8–38.7; range: 0–81.0) for AZT+3TC+NVP; 12.0 months (IQR: 2.3–29.2; range: 0–77.5) for TDF+3TC/FTC+EFV; and, 16.3 months (IQR: 3.7–35.6; range: 0–77.7) for TDF+3TC/FTC+NVP (p = 0.001; Fig 4).

### Baseline Clinical Characteristics

**Fig 5:**
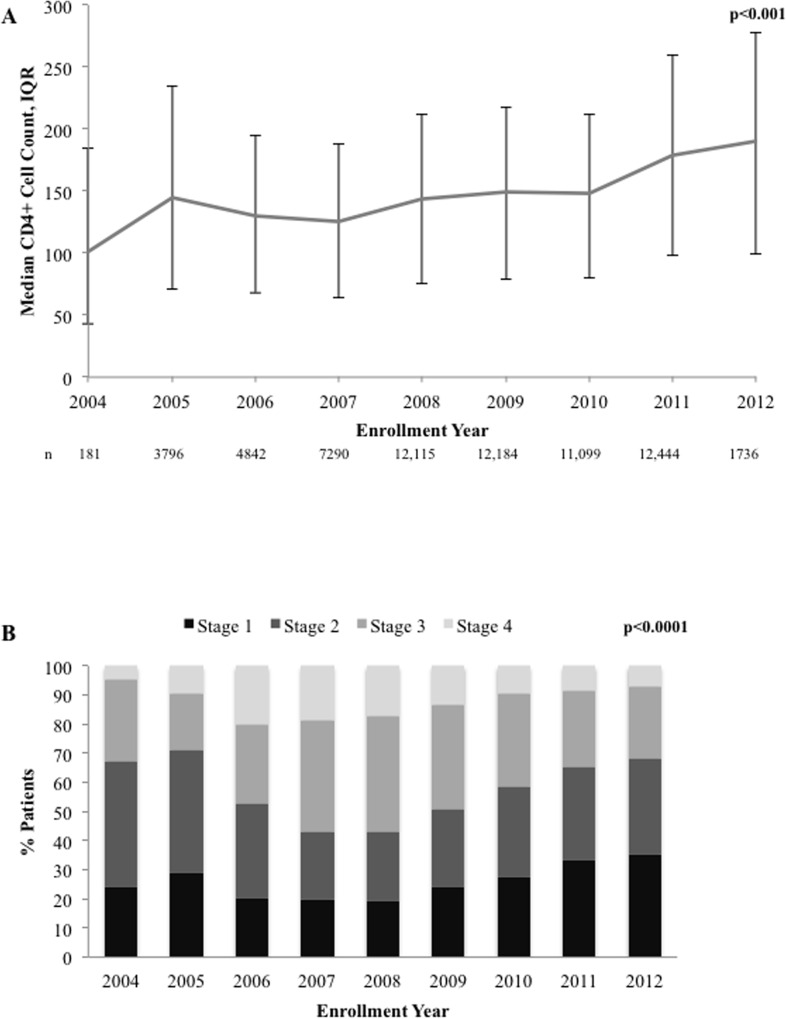
Baseline patient status by enrollment year, ARV-naïve patients. A) median baseline CD4+cell count (cell/mm^3^); and, B) percentage patients by WHO clinical stage.

Of the 70,002 ARV-naïve patients, 59,096 (84.4%) had a documented WHO Stage at ART initiation, of which more than half were asymptomatic or relatively free of symptoms (32,230; 54.5%) meeting the WHO Stage I or II definition. Amongst the naïve patients, 10.2% were HBV-positive, 3.6% had documentation of HCV, 8.9% had evidence of severe anemia, and 20.4% had TB at time of ART initiation. Laboratory measurements indicated that the median baseline CD4+ cell count was 149 cells/mm^3^ (IQR: 78–220) and the median baseline HIV RNA load was 71,310 copies/mL (IQR: 13,293–260,000). Evaluation of baseline CD4+ cell count and WHO stage by enrollment year revealed an overall increasing trend in median baseline CD4+ cell counts between 2004–2012 (p_trend_<0.001; Fig 5A) and trend of increasing percentage of patients who initiated ART with WHO clinical stage 1 or 2 between 2008–2012, respectively (p<0.0001; Fig 5B). Of the ART-naïve patients, 1,231 (1.8%) patients were reported as having died, 21,096 (30.1%) were lost to follow-up, and 96 (0.1%) withdrew from therapy. The majority of patients lost to follow-up were lost within 6 months of ART initiation; of the total 70,002 ART-naïve patients enrolled during the study period, 17.7% were lost by month 6, 21.5% by month 12, 25.9% by month 24, 28.2% by month 36, 29.3% by month 48, 29.8% by month 60, 30.1% by month 72, and 30.1% by month 84.

### Immune Recovery

**Fig 6:**
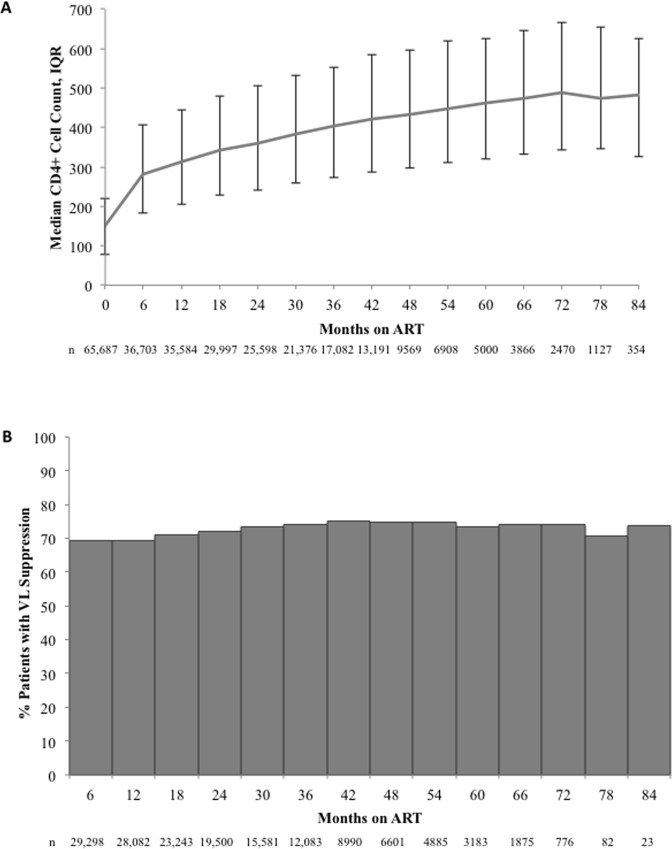
Clinical and virologic outcomes over duration on ART, ARV-naïve patients. A) median CD4+ cell count (cells/mm^3^) over time on ART; and, B) percentage of patients with virologic suppression (≤400 copies/mL) over time on ART.

Median CD4+ cell count for the cohort increased continually over duration on ART from 149 cells/mm^3^ (IQR: 78 – 220) at ART initiation to 487 cells/mm^3^ (IQR: 343–666) at 72 months, after which it appeared to plateau (Fig 6A). The greatest gain was from 0 to 6 months when the median CD4+ cell count gain was 130 cells/mm^3^ (IQR: 57 – 223, n = 35,292); the value differed by baseline CD4+ cell count: 120 cells/mm^3^ (IQR: 63–196) for patients with a baseline CD4+ cell count ≤100 cells/mm^3^ as compared to 140 cells/mm^3^ (IQR: 59–240) for those with baseline 101–200 cells/mm^3^ and (p<0.0001; Table 2). The median change in CD4+ cell count was lowest in those with the highest baseline CD4+ cell count.

**Table 2:** Immunologic, virologic and adherence patterns by baseline CD4+ cell count, previously ARV-naïve patients (N = 70,002).

	Baseline CD4+ Cell Count, cells/mm^3^					p-value
Outcome/pattern evaluated	≤100	101–200	201–350	351–500	>500	
CD4+ cell count change by month 6, cells/mm^3^ (IQR)	120	140	140			<0.0001
	(63–196)	(66–234)	(47–251)			
% with viral suppression^*^ by month 6	65.4	70.7	73.3	71.4	64.4	<0.001
% with viral suppression^*^ by month 12	72.3	77.0	79.3	78.4	73.1	<0.001
% with average adherence ≥95% at month 6	63.1	62.9	64.2	56.5	52.5	<0.001
% with average adherence <50% at month 6	6.4	6.5	6.3	11.7	14.3	<0.001

*Viral suppression defined as VL≤400 cp/mL

### Virologic Suppression & Rebound

Among the patients that were retained beyond 6 months (n = 49,114) and had VL measured at month 6 post-initiation (n = 29,298; 58.9%), 69.3% (n = 20,303) were considered to have achieved early viral suppression (i.e., suppression by month 6). Of the patients that failed to achieve early viral suppression, an additional 14,233 of the patients with subsequent VL data eventually suppressed VL, resulting in an overall 85.4% (n = 34,536/40,418) that achieved viral suppression ever. Using a longitudinal view, suppression rates for the study population gradually increased to 75.0% at 42 months and appeared to plateau through to month 84 (Fig 6B). Using a viral rebound definition of two VLs >400 copies/mL or one VL >5,000 copies/mL, of the patients that achieved early viral suppression, 21.0% (n = 4,262) experienced a viral rebound. Of the patients that experienced the viral rebound following early viral suppression, 60.3% (n = 2,570) re-suppressed.

We also examined the association between baseline CD4+ cell counts and viral suppression at month 6 and month 12 post-initiation of ART. We found that of the patients initiating ART with CD4+ cell counts ≤100 cells/mm^3^, 65.4% were suppressed at month 6 and 72.3% by month 12. Comparatively, for those starting at 101–200 cells/mm^3^, 70.7% were suppressed by month 6 and 77.0% by month 12, and for those starting at 201–350 cells/mm^3^, 73.3% were suppressed at month 6 and 79.3% by month 12. Interestingly, after CD4+ cell counts of 350 cells/mm^3^, we saw a declining trend as the baseline CD4+ cell counts increased, where of those with 351–500 cells/mm^3^, 71.5% were suppressed at month 6 and 78.5% by month 12 and of those with >500 cells/mm^3^ at baseline, only 64.6% were suppressed at month 6 and 73.0% by month 12 (Table 2).

### Treatment Failure by Immunologic & Virologic Criteria

**Fig 7:**
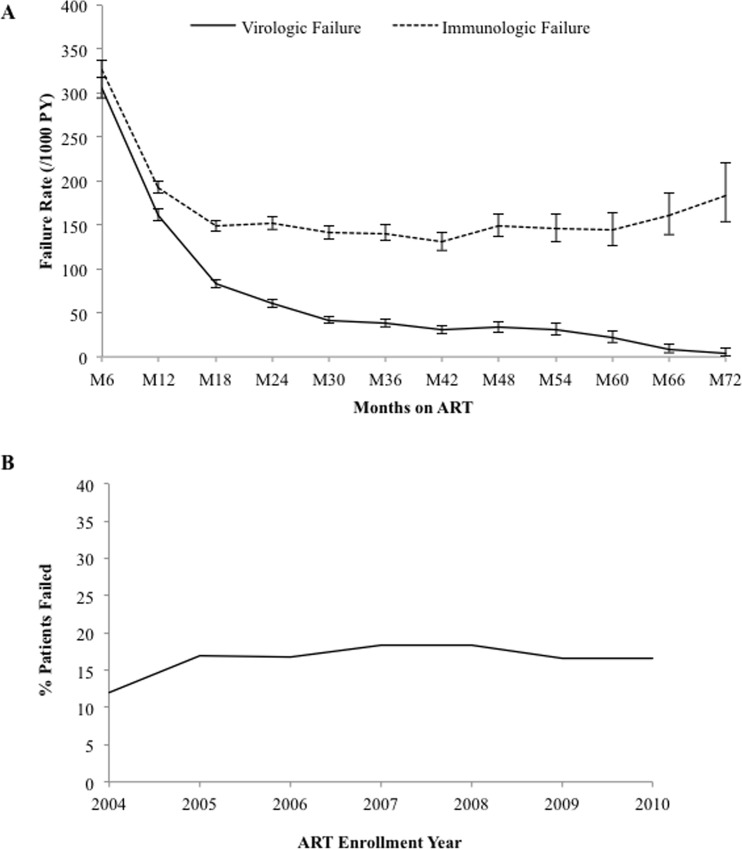
Characterization of treatment failure over time. A) Virologic and immunologic failure rates over duration on ART, 95% CI; and, B) Percentage of patients with virologic failure within first 18 months on ART by enrollment year.

In total, of 30,792 patients eligible to be assessed for VF, 7,504 (24.4%) patients met criteria for failure resulting in an overall VF rate of 96.5 cases per 1000 PY (IQR, 94.3–98.7). The median time to VF was 11.6 months (IQR: 7.6–19.8) and rate of VF was highest at month 6 at 306.0 cases per 1000 PY (IQR, 294.5–318.0), then decreased to 30.9 cases per 1000 PY (IQR, 26.5–35.9) at month 42 at which point it appeared to plateau (Fig 7A). Of the 45,130 total patients eligible to be assessed for IF, 15,353 (34.0%) met criteria for failure, resulting in an overall IF rate of 182.1 cases per 1000 PY (IQR: 179.2 – 185.0). The median time to recorded IF was 16.0 months (IQR: 8.8–28.7) and rates were also highest at month 6 at 327.5 cases per 1000 PY (IQR, 317.4–337.9) but plateaued early at month 18, after which it hovered between 130 to 152 cases per 1000 PY from month 24 to month 60. Over 50% of all failures occurred within the first 12 months of ART for VF, and within the first 18 months of ART for IF. In assessing the predictive power of immunologic criteria for detecting failure as compared to virologic criteria, we found a 60.9% sensitivity, 69.9% specificity, 37.1% positive predictive value, and 86.0% negative predictive value. No notable trend was seen in percentage of patients with VF within the first 18 months by enrollment year (Fig 7B).

In bivariate analyses, age, education, employment status, marital status, ART regimen, ART enrollment year, site type, WHO stage, BMI, CD4+ cell count, HBV and HCV status, VL, anemic state, adherence were all found to be predictors of VF (Table 3). Following multivariate adjustments, factors that remained associated with VF in the model with multiple imputations were age, ART regimen, CD4+ cell count, VL, and adherence. Baseline BMI and ART enrollment year were of borderline significance in the complete case model, but the variables were no longer significant following imputation. In the final model, the risk for VF was greater for patients who were younger, on regimens other than TDF+EFV, had lower CD4+ cell counts (both baseline and at prior visit), higher baseline VL, unsuppressed VL at current visit, and lower percent ART adherence during the period preceding the visit. Our data indicate that for this cohort, those on NVP regimens generally were at higher risk for VF than those on equivalent EFV regimens. Patients on TDF+FTC/3TC+NVP, AZT+3TC+EFV, AZT+3TC+NVP, or d4T+3TC+NVP patients were at higher risk of VF than those on TDF+FTC/3TC+EFV (aHR: 1.57, 1.24, 1.37, 1.55, respectively).

**Table 3:** Predictors of virologic and immunologic failure for ARV-naïve patients.

Predictors	Virologic Failure			Immunologic Failure		
	Bivariate Analysis	Complete	Multiple Imputations**	Bivariate Analysis	Complete	Multiple Imputations**
		Cases**			Cases**	
	(n = 30,792)	(n = 19,454)	(n = 30,792)	(n = 45,130)	(n = 35,836)	(n = 45,130)
	HR (95% CI)	aHR (95% CI)	aHR (95% CI)	HR (95% CI)	aHR (95% CI)	aHR (95% CI)
**Age** (vs. 15–29 yrs)						
30–34 years	0.80 (0.72–0.88)	0.87 (0.81–0.94)	0.86 (0.81–0.92)	0.90 (0.87–0.94)		
35–40 years	0.75 (0.70–0.81)	0.87 (0.82–0.93)	0.84 (0.79–0.89)	0.93 (0.88–0.98)		
41+ years	0.67 (0.60–0.74)	0.79 (0.72–0.86)	0.78 (0.73–0.84)	0.88 (0.83–0.94)		
**Sex** (Female vs. Male)	1.00 (0.96–1.04)			0.90 (0.88–0.93)	0.84 (0.79–0.90)	0.86 (0.82–0.90)
**Education** (vs. none/primary)						
Secondary	0.95 (0.90–1.01)			0.96 (0.89–1.04)		
Tertiary	0.84 (0.79–0.89)			0.87 (0.81–0.94)		
**Employment status**						
Laborer/service/admin	0.87 (0.82–0.93)			0.96 (0.91–1.02)		
Manager/professional	0.78 (0.70–0.86)			0.84 (0.78–0.89)		
**Marital status** (vs. single)						
Married	0.86 (0.80–0.93)			0.94 (0.91–0.98)	0.92 (0.87–0.97)	0.94 (0.90–0.98)
Separated/Divorced	1.01 (0.87–1.16)			0.98 (0.89–1.07)	0.96 (0.86–1.07)	0.98 (0.89–1.07)
Widowed	0.88 (0.78–0.99)			0.90 (0.84–0.97)	0.95 (0.89–1.01)	0.96 (0.90–1.02)
**HIV risk factor** (heterosexual sex vs. other)						
	1.00 (0.90–1.11)			1.00 (0.92–1.09)		
**ART enrollment year**						
2007–09 vs. 2004–06	0.83 (0.63–1.10)	1.12 (1.03–1.22)	0.93 (0.81–1.06)	1.13 (0.76–1.67)	1.25 (1.06–1.47)	1.19 (0.99–1.44)
2010–11 vs. 2004–06	0.77 (0.48–1.24)	1.37 (1.13–1.66)	0.94 (0.78–1.13)	1.25 (0.81–1.94)	1.36 (1.10–1.67)	1.34 (1.06–1.69)
**Tertiary Site** (vs secondary)	0.72 (0.55–0.95)			0.87 (0.69–1.09)		
**ART regimen** (vs. TDF+FTC/3TC+EFV)						
TDF+FTC/3TC+NVP	1.68 (1.43–1.97)	1.46 (1.26–1.68)	1.57 (1.35–1.83)	1.16 (1.06–1.27)	1.40 (1.23–1.58)	1.29 (1.15–1.44)
AZT+3TC+EFV	1.34 (1.14–1.59)	1.18 (1.05–1.33)	1.24 (1.12–1.37)	1.00 (0.92–1.10)	0.98 (0.87–1.09)	0.98 (0.91–1.06)
AZT+3TC+NVP	1.34 (1.13–1.59)	1.30 (1.10–1.54)	1.37 (1.20–1.55)	0.95 (0.83–1.09)	1.12 (1.01–1.25)	1.07 (0.96–1.18)
D4T+3TC+EFV	1.52 (1.21–1.92)	1.35 (0.96–1.89)	1.17 (0.95–1.43)	0.93 (0.77–1.11)	0.87 (0.69–1.09)	0.88 (0.71–1.09)
D4T+3TC+NVP	1.68 (1.25–2.27)	1.52 (1.25–1.83)	1.55 (1.37–1.76)	1.12 (0.74–1.69)	1.36 (1.19–1.56)	1.21 (1.05–1.39)
**WHO stage (vs. 1)**						
2	1.13 (1.01–1.27)			1.03 (0.96–1.10)		
3	1.25 (1.09–1.43)			1.10 (0.93–1.30)		
4	1.54 (1.32–1.81)			1.14 (0.95–1.36)		
**TB co-infection**	1.00 (0.89–1.11)			0.96 (0.89–1.03)		
**HBV co-infection**	1.10 (1.02–1.18)			1.15 1.07–1.23)		
**HCV co-infection**	0.90 (0.80–1.01)			0.92 (0.82–1.03)		
**Baseline BMI** (vs. normal)						
Underweight	1.24 (1.14–1.34)	1.05 (0.95–1.16)	1.03 (0.97–1.09)	1.10 (1.01–1.20)		
Overweight	0.90 (0.85–0.96)	1.07 (1.01–1.13)	1.03 (0.98–1.10)	0.93 (0.89–0.98)		
**Baseline CD4 count, cells/mm**^**3**^						
101–200 vs. ≤100	0.73 (0.67–0.79)	0.82 (0.76–0.89)	0.82 (0.77–0.88)	0.93 0.86–0.99)	0.99 (0.89–1.12)	1.13 (1.04–1.22)
201–350 vs. ≤100	0.58 (0.53–0.63)	0.71 (0.64–0.78)	0.67 (0.62–0.73)	1.24 (1.13–1.35)	1.43 (1.26–1.61)	1.68 (1.50–1.89)
>350 vs. ≤100	0.70 (0.57–0.85)	0.78 (0.66–0.93)	0.72 (0.61–0.86)	3.29 (3.01–3.60)	3.56 (3.13–4.04)	4.39 (3.84–5.03)
**Baseline Viral load, copies/mL**						
10,001−10^5^ vs. ≤10^4^	1.19 (1.12–1.26)	1.06 (0.96–1.17)	1.14 (1.07–1.22)	0.83 (0.80–0.87)	0.89 (0.82–0.97)	0.96 (0.90–1.03)
>10^5^ vs. ≤10^4^	1.35 (1.22–1.50)	1.16 (1.09–1.23)	1.22 (1.15–1.30)	0.89 (0.84–0.94)	0.93 (0.86–1.00)	1.00 (0.95–1.06)
**Baseline Anemia** (vs. no anemia)						
Mild/moderate anemia	1.21 (1.11–1.31)			1.09 (0.94–1.26)	1.07 (1.01–1.12)	1.04 (1.00–1.09)
Severe anemia	1.35 (1.08–1.68)			1.15 (0.90–1.47)	1.08 (0.99–1.18)	1.06 (0.99–1.13)
BMI^*^						
Underweight vs. normal	1.18 (0.99–1.40)			1.28 (1.20–1.37)	1.16 (1.07–1.27)	1.15 (1.06–1.25)
Overweight vs. normal	0.82 (0.71–0.94)			0.85 (0.82–0.88)	0.94 (0.88–1.00)	0.91 (0.88–0.95)
**CD4 count, cells/mm**^3^^*^						
101–200 vs. ≤100	0.37 (0.33–0.40)	0.55 (0.48–0.63)	0.64 (0.56–0.73)	0.72 (0.63–0.82)	0.74 (0.65–0.84)	0.61 (0.55–0.68)
201–350 vs. ≤100	0.22 (0.19–0.25)	0.47 (0.39–0.57)	0.52 (0.46–0.60)	0.44 (0.36–0.53)	0.44 (0.37–0.53)	0.37 (0.33–0.41)
>350 vs. ≤100	0.16 (0.13–0.19)	0.44 (0.32–0.59)	0.47 (0.37–0.59)	0.43 (0.35–0.54)	0.40 (0.32–0.49)	0.27 (0.24–0.31)
Viral load >1000 copies/mL^*^	77.74 (57.8–104.5)	68.05 (48.59–95.31)	38.13 (25.70–56.56)	3.32 (2.75–3.99)	3.20 (2.58–3.98)	2.11 (1.81–2.45)
Anemia^*^						
Mild/moderate vs. no anemia	1.33 (1.25–1.43)			1.31 (1.13–1.51)	1.28 (1.15–1.42)	1.24 (1.16–1.32)
Severe vs. no anemia	1.94 (1.72–2.18)			1.90 (1.60–2.24)	1.54 (1.27–1.88)	1.60 (1.36–1.90)
**Avg % adherence** (vs. ≥95%)^*^						
80–94.9%	1.50 (1.36–1.67)	1.31 (1.24–1.39)	1.34 (1.20–1.49)	1.19 (1.10–1.29)	1.20 (1.11–1.30)	1.18 (1.10–1.26)
<80%	3.01 (2.60–3.50)	1.54 (1.43–1.65)	1.81 (1.56–2.11)	2.02 (1.76–2.32)	1.40 (1.23–1.58)	1.68 (1.47–1.92)

* BMI, CD4 count, viral load, anemia, and average % ART adherence are time-updated covariates except where noted as baseline; all other predictors are at baseline.

** The final multivariate model is presented, which accounted for correlation within sites using clustered robust standard errors.

Similar to VF, the strongest predictors of IF in the adjusted model with multiply imputed data were lower CD4+ cell count and unsuppressed VL at previous visit, and poorer adherence. Lower BMI and anemia at previous visit were also predictive of IF. In measuring IF, female sex was slightly protective as was being married (versus single). Patients on NVP-containing regimens paired with TDF or d4T also appeared to be at a slightly higher risk for IF as compared to those on TDF+EFV. Unlike what was seen for VF, higher baseline CD4+ cell counts and later enrollment year were associated with higher risk for IF in both complete case and multiple imputation adjusted Cox regression models.

### Longitudinal Adherence Patterns

**Fig 8:**
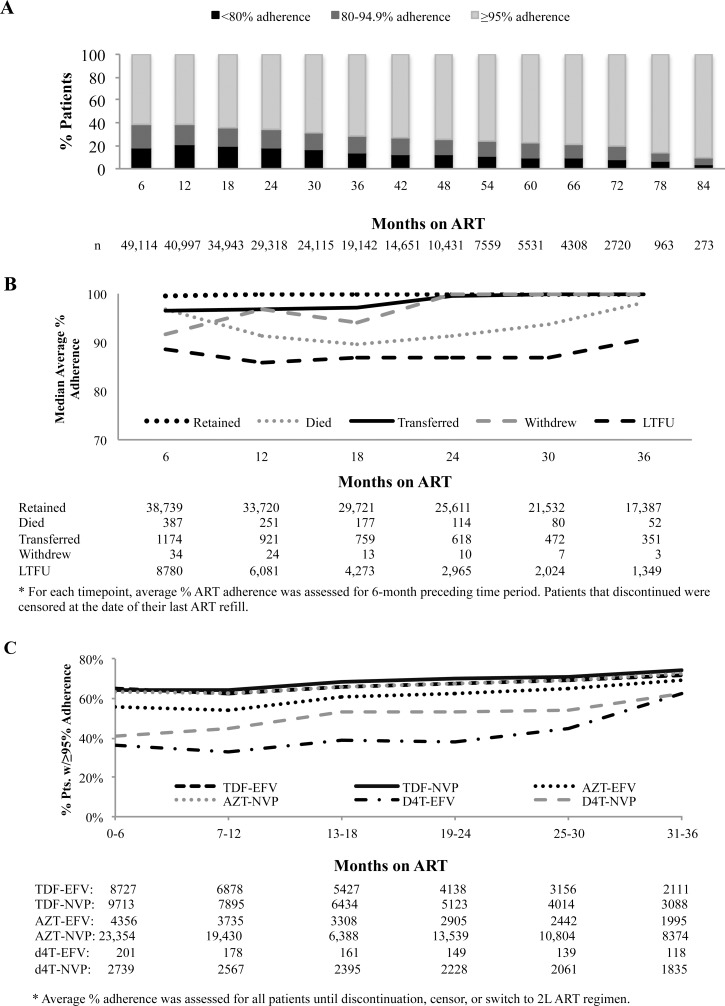
Long-term ART adherence for previously ARV-naïve patients on ART >6 months (N = 49,114). A) All patients on ART; B) Median percent ART adherence by discontinuation group; and, C) Percent of patients with ≥95% adherence over time by starting ART regimen.

We were able to examine long-term adherence patterns for patients that were retained as long as 84 months post-initiation of ART, with over 5,500 people on ART for over 5 years. We found that for those that remained in the program or had sufficient years of follow-up time since enrollment, the majority exhibited very strong (i.e., ≥95%) adherence for many years (Fig 8A).

To examine if adherence patterns appear different in patients that were retained long-term versus those that eventually discontinued treatment, we compared the median percent adherence for each 6-month time interval up to month 36 post-initiation of ART for patients who were retained on ART versus those that eventually transferred, died, withdrew or were lost to follow-up. For those that were retained or transferred to another site, the median percent adherence was above 95% during 0–6 months and rose to 100%. For those that eventually died, median adherence also started above 95%, but dropped gradually over time on ART and rose again in those that died closer to 30 months. Finally, for patients that were eventually LTFU or withdrew, median percent adherence began at 88.5% and never rose above 95% during the observation period (Fig 8B). We also examined association between initial ART regimen and adherence and found that patients on d4T-containing regimens had a lower percentage of patients that retained a ≥95% average adherence over time as compared to those on TDF- or AZT-containing regimens (Fig 8C).

We also examined the association between baseline CD4+ cell count and early adherence patterns (i.e., first 6 months on ART). We found that of the patients with baseline CD4+ cell counts ≤350 cells/mm^3^, 63.3% had an average adherence of ≥95% during their first 6 months, where adherence patterns were not significantly different when CD4+ cell count groups were further stratified into ≤100 cells/mm^3^, 101–200 cells/mm^3^, and 201–350 cells/mm^3^ categories (63.1%, 62.9%, 64.2%, respectively; Table 2). However, we did find that of patients with baseline CD4+ cells counts from 351–500 cells/mm^3^, 56.5% had ≥95% adherence by month 6 and of those with baseline counts >500 cells/mm^3^, only 52.5% had ≥95% adherence by month 6. Additionally, of those with baseline counts of >500, 14.3% had an average adherence of ≤50% as compared to 11.7% of those with baseline counts from 351–500 cells/mm^3^ and 6.4% with counts ≤350 cells/mm^3^; these differences were statistically significant (p<0.001; Table 2).

## Discussion

In this evaluation, we examined long-term outcomes for a large cohort of adult patients enrolled in the Harvard/APIN PEPFAR program in Nigeria between the years 2004–2012. As a part of these evaluations, we examined longitudinal patterns in ART regimen prescription patterns, long-term immunologic and virologic outcomes, adherence patterns, and continuous predictors of treatment failure. Overall, we report strong long-term outcomes, consistent with data from earlier short- and medium-term evaluations from other international scale-up programs in South Africa, Ethiopia, Uganda, Botswana, Senegal and China [4,5,7,9,31,32].

In the past decade, the recommended drugs and regimens for optimal ART were changing worldwide. The patents for many of the early antiretrovirals (ARVs) were expiring and many generic versions were being manufactured in India, Thailand, Brazil, and elsewhere. In addition to less expensive versions of these drugs, FDCs were made available to reduce the pill burden and improve adherence. The PEPFAR program provided important assistance in expediting the U.S. Food and Drug Administration review and approval of these drugs [33]. As a result, by 2008, the cost of drugs for ARVs dropped to levels well below US$300 per year. Despite these advances, however, the newer and more efficacious drugs being developed in the United States and Europe remain prohibitively expensive and are still not included in most ART programs in Africa, leaving the depth of the pharmacy comparatively shallow. As such, this evaluation of outcomes focuses on a limited set of 1L regimens. Our data indicated that during the early years of enrollment, the majority of patients were on d4T-containing 1L regimens. Over the years, d4T was phased out, with AZT-containing regimens slowly predominating and a gradual increasing percentage of patients being initiated on TDF-containing regimens.

Despite the low variety in types of ARVs, as the general availability of ART rapidly increased with scale-up of PEPFAR and Global Fund supported programs and the WHO recommendations for initiating ART evolved over these years, increasing CD4 count criteria from 200 to 350 cells/mm^3^, we hypothesized that the baseline clinical characteristics of newly enrolled patients changed over the enrollment years. As expected, this study revealed higher baseline CD4+ cell counts and greater proportion of patients enrolling at lower WHO stages in the program from 2004–2012. As the years passed, and the sicker patients enrolled on ART during the early years of the program, the patients enrolled in later years typically were initiated at higher baseline CD4+ cell counts. Of note, in 2013, the WHO recommended that ART should be initiated in all individuals with a CD4 count >350 and ≤500 cells/mm^3^. Additional evaluations on data beyond 2013 would be expected to reveal even higher median CD4+ cell counts.

While the enrolled patient population started with relatively low median CD4+ cell counts at ART initiation, the rate of immune recovery was strong up through 84 months of follow-up. Viral suppression rates were also high out to 84 months of follow-up, mimicking the findings from some recent, smaller middle- and long-term outcome analyses conducted in Botswana, South Africa, Senegal, and Uganda [4,31,32,34].

A study by Lima *et al*., from British Columbian, Canada, indicated that patients that started ART with CD4+ cell counts of at least 500 cells/mm^3^ were more likely to be virally suppressed at 9 months post-initiation that than those with lower starting CD4+ cell counts [11]. In our evaluation, we found that patients that initiated ART with baseline CD4+ cell counts between 201–350 cells/mm^3^ were more likely than those with lower and higher baseline CD4+ cell counts to be suppressed at both month 6 and month 12 post-initiation of ART. From a clinical perspective, it makes sense that patients starting with the lower CD4+ cell counts would need more time to achieve viral suppression. However, for the groups starting with higher CD4+ cell counts, it is possible that they were feeling well at the time they started medication and perceived a lower benefit to taking all of their medications or maintaining a high level of adherence. Interestingly, we also found that early average adherence was lower for those with baseline CD4+ cell counts >350 cells/mm^3^ as compared to those patients with counts ≤350 cells/mm^3^. Furthermore, we found a higher relative hazard of virologic failure for patients with baseline CD4 >350 cells/mm^3^ compared with those with baseline CD4 of 201–350 cells/mm^3^. This finding is consistent with those of Grimsrud et al, who found that patients with baseline CD4 counts≥300 cells/μL were more likely to be LTFU after 24 months on ART than those patients with CD4 counts of 150–199 μL [35]. Additional studies should be conducted to better understand the associations between higher baseline CD4+ cell counts and subsequent viral suppression rates, particularly as countries are moving towards large-scale roll-out of test and treat programs.

The regular measurement of VLs was a unique feature of our program, allowing for assessment of VF, the most objective determination of ART outcome as would be measured in resource-rich settings. The baseline risk factors for VF we found (younger age, lower CD4 count, and higher VL) and the time-updated risk factors (lower CD4 count, unsuppressed VL, and poorer adherence) should be useful in identifying higher risk patients for targeted adherence counseling and monitoring strategies. While one recent study of long-term outcomes in Cameroon showed increased risk for VF in males versus females [6], in our analyses of over 30,000 patients, we were not able to corroborate the finding similar to another recent study from Uganda [7]. The increased risk for VF for patients on NVP regimens compared to EFV regimens for all three NRTIs suggests that NVP regimens should be prescribed cautiously when appropriate. Scarsi *et*. *a*l. showed that patients who initiated ART on TDF in combination with NVP had higher risk of VF than patients who initiated on AZT with NVP in our program [36], which our data also show. Additionally, our data indicate that patients who initiated on TDF in combination with EFV had lower risk for VF than those on AZT with EFV, which has previously been shown in a randomized clinical trial [37]. Our findings support the view that NNRTI effectiveness differs by NRTI selection, possibly due to drug-drug interactions [36], and are consistent with the WHO recommendations that EFV is used as the preferred NNRTI in a patient newly initiated ART [38].

Interestingly, unlike for VF, higher baseline CD4+ cell counts were associated with IF; this may be because those with higher baseline CD4+ cell counts are more likely to have their CD4+ cell count drop below baseline, one of the criteria for immunologic failure, during ART than those with already low baseline levels. This may also explain why later enrollment year was also weakly associated with higher risk of immunologic failure as the baseline CD4+ cell count criteria for ART enrollment increased in 2010.

Just as our previous evaluation showed that the largest percentage of patient LTFU occurred during the first 12–18 months of ART [39], we found that risk of virologic failure was also highest in the first year on ART and decreased with longer duration on ART. Thus, the first year of ART, and particularly the first few visits, is a crucial time for adherence counseling and monitoring adherence directed interventions [5,39]. ART knowledge among patients and health care workers, drug adherence counseling, and patient monitoring are crucial for optimization of patient outcomes.

Because we had both VF and IF data, we were able to examine accuracy of IF criteria to predict VF in a large cohort of patients. Similar to the earlier study by Rawizza et al. [40], but now in a larger cohort with additional years of data, we can re-confirm that IF criteria is not a strong predictor of VF and that VF criteria detect failure almost 5 months earlier than IF criteria. These findings are important to consider in making decisions about treatment switch and have implications when considering development of drug resistance mutations with each additional month on a failing drug regimen.

Our study also demonstrated strong long-term adherence, similar to what was found in a smaller, middle-term ART cohort study from Botswana [31], further indicating that sustained, long-term positive outcomes are possible in sub-Saharan African settings. Interestingly, we found an association between higher baseline CD4+ cell counts and decreased adherence; it might be speculated that patients that felt healthier might be less likely to adhere. These data are consistent with the associations previously noted between adherence and viral suppression as well as adherence and immunologic failure.

This evaluation does have some limitations. As a retrospective observational cohort study, this analysis was limited by missing data, which was expected considering the rapid scale-up of the program and data collection in the context of routine care. While we attempted to control for some of the missing data issues through use of multiple imputation methods, it is possible that patients who were missing certain time-updated clinical laboratory values may have been more likely to develop failure than those who were not missing data, which may have affected the imputation results and the magnitudes of association in the multivariate models. The study was limited because the program did not actively trace all patients that were lost, which likely resulted in an overestimation of LTFU and underestimation of mortality, transfers and withdrawals as has been indicated in previous other studies in which LTFU patients were traced [41–47]. The VF analysis only included patients who were retained on ART and for whom we could evaluate VF using the program definition and does not include those with fewer than two VLs; thus, our failure rates do not include patients who were lost to follow-up or who had fewer than two VLs, which are in themselves problematic outcomes for large-scale ART programs. Also, we censored failure patients at their first failing VL or switch to 2L ART, and did not assess substitutions to other 1L ART regimens or second failures, which could provide useful insight. Finally, as this was an observational cohort study, we were not able to control for all factors related to virologic failure and there is residual confounding that must be considered in interpretation of the regimen comparison data.

This evaluation also has notable strengths. The large enrollment of the program and lengthy follow-up provide statistical strength to reinforce our findings. To our knowledge, other large cohorts examining ART outcomes include one from Botswana, which described medium-term outcomes for over 126,000 patients [48] and another multi-cohort study that presented medium-term retention data for over 130,000 patients across Africa and Asia [3]. Additionally, a recent meta-analysis consolidate information on multiple short-, medium- and long-term analyses, but none with over 70,000 patients, where at least 5,500 have over 5-years of ART outcome data [49]. Furthermore, our continual 6-month measurements for critical variables enabled us to correlate VF with these risk factors as they changed over time. Unlike other evaluations of long-term clinical outcomes, this analysis included time-updated clinical and adherence data, which we found to be better predictors of treatment failure, whether measured by virologic or immunologic criteria, than the baseline measurements alone.

## Conclusion

The United Nations Sustainable Development Goals (SDGs) aims to see an end to the HIV/AIDS epidemic by 2030. While access to ART rose dramatically between 2004 to 2014 as a result of major contributions from the massive scale-up programs supported by PEPFAR, the Global Fund for Tuberculosis, AIDS and Malaria, the World Bank, the Clinton Foundation, it was estimated in 2013 that only 20% of patients in Nigeria that required ART were actually receiving it [1]. Clearly, much remains to be done if the SDGs are to be achieved in Nigeria by 2030. The progress thus far and positive long-term outcomes for patients that initiated ART suggest, however, that these ambitious goals are achievable.
